# Extracellular membrane vesicles from umbilical cord blood-derived MSC protect against ischemic acute kidney injury, a feature that is lost after inflammatory conditioning

**DOI:** 10.3402/jev.v2i0.21927

**Published:** 2013-12-10

**Authors:** Lotta Kilpinen, Ulla Impola, Lotta Sankkila, Ilja Ritamo, Maria Aatonen, Sami Kilpinen, Jarno Tuimala, Leena Valmu, Jouko Levijoki, Piet Finckenberg, Pia Siljander, Esko Kankuri, Eero Mervaala, Saara Laitinen

**Affiliations:** 1 Finnish Red Cross Blood Service, Helsinki, Finland; 2 Division of Biochemistry and Biotechnology, Department of Biosciences, University of Helsinki, Helsinki, Finland; 3 MediSapiens Ltd., Helsinki, Finland; 4 Orion Pharma Ltd., Espoo, Finland; 5 Department of Pharmacology, Institute of Biomedicine, University of Helsinki, Helsinki, Finland; 6 Division of Pharmaceutical Biosciences, Faculty of Pharmacy, University of Helsinki, Helsinki, Finland

**Keywords:** stem cells, cell therapy, inflammation, immunology, membrane trafficking

## Abstract

**Background:**

Mesenchymal stromal cells (MSC) are shown to have a great therapeutic potential in many immunological disorders. Currently the therapeutic effect of MSCs is considered to be mediated via paracrine interactions with immune cells. Umbilical cord blood is an attractive but still less studied source of MSCs. We investigated the production of extracellular membrane vesicles (MVs) from human umbilical cord blood derived MSCs (hUCBMSC) in the presence (MVstim) or absence (MVctrl) of inflammatory stimulus.

**Methods:**

hUCBMSCs were cultured in serum free media with or without IFN-γ and MVs were collected from conditioned media by ultracentrifugation. The protein content of MVs were analyzed by mass spectrometry. Hypoxia induced acute kidney injury rat model was used to analyze the in vivo therapeutic potential of MVs and T-cell proliferation and induction of regulatory T cells were analyzed by co-culture assays.

**Results:**

Both MVstim and MVctrl showed similar T-cell modulation activity *in vitro*, but only MVctrls were able to protect rat kidneys from reperfusion injury in vivo. To clarify this difference in functionality we made a comparative mass spectrometric analysis of the MV protein contents. The IFN-γ stimulation induced dramatic changes in the protein content of the MVs. Complement factors (C3, C4A, C5) and lipid binding proteins (i.e apolipoproteins) were only found in the MVctrls, whereas the MVstim contained tetraspanins (CD9, CD63, CD81) and more complete proteasome complex accompanied with MHCI. We further discovered that differently produced MV pools contained specific Rab proteins suggesting that same cells, depending on external signals, produce vesicles originating from different intracellular locations.

**Conclusions:**

We demonstrate by both *in vitro* and *in vivo* models accompanied with a detailed analysis of molecular characteristics that inflammatory conditioning of MSCs influence on the protein content and functional properties of MVs revealing the complexity of the MSC paracrine regulation.

Extracellular membrane vesicles (MVs) were first identified from platelets as “platelet dust” 50 years ago (1) and their importance in platelet function was largely ignored for decades. Currently, secreted MVs are well established and their role in cellular communication has been confirmed by many studies, in which MVs have been shown to transfer surface receptors, mRNA, miRNA and signalling molecules, such as bioactive lipids (2–6). As suggested by Thery et al. (7), we use the term extracellular MVs to include all secreted cellular MVs such as exosomes, microvesicles and apoptotic bodies.

Mesenchymal stromal cells (MSCs) have been shown to have therapeutic potential in many immunological disorders including graft-versus-host disease (8), Crohn's disease (9) and rheumatoid arthritis (10). An increasing number of *in vitro* studies have shown that MSCs have the capability to affect both innate and adaptive immune response. Specifically, MSCs are able to directly inhibit T-cell proliferation, change the T-helper lymphocyte balance and induce regulatory T-cells (Tregs) (11–13). The mechanisms by which MSCs exert their immunomodulatory effects are still largely unknown, but there is an increasing amount of evidence suggesting that the therapeutic effects are mediated by paracrine factors, such as the tryptophan-degrading enzyme indoleamine 2,3-dioxygenase (IDO) and prostaglandin E_2_ (PGE_2_) (11, 14, 15). Recent reports show that cell-derived extracellular MVs are responsible for at least part of the therapeutic paracrine function of these cells (16–18).

Ischemia/reperfusion injury (IRI) is a major cause of clinical acute kidney injury (AKI). The pathophysiology consists of alterations in the renal hemodynamics, inflammatory response, kidney endothelial and tubular cell injuries followed by a repair process (19, 20). Several studies have shown that T-cells are the key mediators in renal IRI (21). However, it has previously been shown that immunosuppressive Tregs mediate at least in part, the renoprotective effects of ischemic preconditioning (22) and participate in the repair of kidney IRI (23). MSCs as such are described to be a new therapeutic tool for AKI (24). In addition to MSCs, MVs secreted by MSCs are also shown to have a protective effect against the ischemia-induced AKI, mediated by transferred mRNA (6, 17, 18, 25). In the site of the inflammation in injured tissues, there is an abundant amount of different cytokine molecules. The surrounding microenvironment plays a major role in regulating the MSC immunoregulatory function. Several studies have demonstrated that cytokines such as interferon-gamma (IFN-γ) and tumour necrosis factor alpha (TNF-α) regulate the production of MSC-derived soluble factors (26–29). Moreover, priming with cytokines has been suggested to be essential for both *in vitro* and *in vivo* immunomodulatory activity of MSCs (30–32).

It has been reported that MSC origin affects their immunomodulatory properties (33–35) and response to the cytokine stimulus (36). In the present study, our aim was to examine the effect of IFN-γ stimulus on the secretion and function of MVs originating from umbilical cord blood-derived MSCs. The protein content of the produced MVs was analyzed in relation to their therapeutic effect in ischemia-induced AKI *in vivo* model. Furthermore, the ability of MVs to induce regulatory T-cells and suppress the proliferation of T-cells was assessed.

## Materials and methods

### Cord blood derived mesenchymal stromal cells

Cord blood units were collected at the Helsinki University Central Hospital, Department of Obstetrics and Gynaecology, and Helsinki Maternity Hospital. All donors gave informed consent and the ethical review board of Helsinki University Central Hospital and the Finnish Red Cross Blood Service approved the study protocol. Human umbilical cord blood-derived MSC (hUCBMSCs) were established with protocol designed in our lab and described elsewhere (37). Briefly, cord blood mononuclear cells were isolated by Ficoll-Paque Plus (GE Healthcare, Uppsala, Sweden) gradient centrifugation. 1×10^6^/cm^2^ mononuclear cells were plated on fibronectin-coated (Sigma-Aldrich, St. Louis, MO) tissue culture plates (Nunc, Roskilde, Denmark) in Minimum Essential Alpha-Medium (α-MEM) with Glutamax™ (Gibco, Paisley, UK), supplemented with 10% foetal bovine serum (Gibco), 10 ng/mL epidermal growth factor (EGF, Sigma-Aldrich), 10 ng/mL recombinant platelet-derived growth factor (rhPDGF-BB R&D Systems, Minneapolis, MN), 50 nM dexamethasone (Sigma-Aldrich), 100 U/mL penicillin (Gibco) and 100 µg/mL streptomycin (Gibco). The cells were allowed to adhere overnight, and non-adherent cells were washed with medium changes. The initial hUCBMSC establishment was performed under hypoxic conditions (5% CO_2_, 3% O_2_ at 37°C). For further experiments, hUCBMSCs were cultured in normoxic conditions (5% CO_2_ and 20% O_2_ at 37°C) and proliferation media was renewed twice a week. The expression of characteristic human MSC markers and adipogenic, chondrogenic and osteogenic differentiation capacity was analyzed (38). See Supplementary file for detailed methods.

### Extracellular MVs

hUCBMSCs were incubated with serum-free starvation medium with or without 100 ng/mL IFN-γ (Sigma-Aldrich) at 37°C with 5% CO_2_, for 24–48 hours and the proliferation and cell morphology was monitored by the investigator. After incubation, the cells and the conditioned media were collected for further analysis. Cell viability was analyzed by flow cytometry using probidium iodide staining (BD Biosciences, San Jose, CA) or a Nucleocounter NC-100 cell counter (Chemometec, Lillerod, Denmark).

To collect MVs, the conditioned medium was first centrifuged at 2,000×*g* for 20 min at +4°C. The supernatant was further ultracentrifuged at 100,000×g for 1–2 hours at +4°C (Sorwall WX Ultra 80, Thermo Fisher Scientific Inc., Waltham, MA), washed with phosphate-buffered saline (PBS) and submitted to a second 1–2 hours ultracentrifugation under the same conditions. MVs were suspended in a small volume of PBS and stored at −80°C. The protein concentration was measured with NanoDrop (Thermo Fisher Scientific Inc.) or with Pierce BCA Protein Assay kit (Thermo Fisher Scientific Inc.).

### Electron microscopy

MV specimen preparation for scanning electron microscopy (SEM) and for negative staining was done by the personnel of the Electron Microscopy Unit, Institute of Biotechnology, and University of Helsinki. SEM was performed with FEI Quanta 250 Field Emission Gun Scanning Electron Microscope and negative stainings with FEI Tecnai 12 Transmission Electron Microscope (Philips Electron Optics, Holland).

### Nanoparticle tracking analysis

After centrifugation, MVs were resuspended with PBS, and analyzed with nanoparticle tracking analysis (NTA) instrument LM14C with blue laser (405 nm, 60 mW, NanoSight Technology, London, UK) and CMOS camera (Hamamatsu Photonics K.K., Hamamatsu City, Japan) to determine the vesicle size distributions and concentrations. Samples were injected manually and data acquisition was done at ambient temperature. Samples were run as triplicates. Settings for data acquisition were: basic, camera level 13, autosettings off, polydispersity and reproducibility high with particles per image 40–100 (acquisition time 90 seconds). Calibration was done by using 0.2-µm Fluoresbrite^®^ Multifluorescent Microspheres (Polysciences, Inc., Warrington, PA). Data were analyzed with NTA 2.3 software with settings expert, background extraction/auto blur/auto-minimum track length on and minimum expected particle size 50 nm.

### MS analysis

Gel-based proteome analysis of MVs was performed with the liquid chromatography–mass spectrometry (LC-MS) of tryptic peptides. Proteins from collected MVs were run in a 4–20% sodium dodecyl sulfate polyacrylamide gel electrophoresis (SDS-PAGE) gel (Bio-Rad Laboratories, Hercules, CA). The gel was silver stained (39) and sliced into pieces. Each gel piece was processed using an in-gel reduction, alkylation and trypsin digestion protocol as previously described (40). Peptides were loaded to precolumn (Protecol Guard C18, 150 µm, 10 mm, 3 µm; SGE Analytical Science Pty Ltd, Melbourne, Australia) and separated in a reversed-phase analytical column (Acclaim PepMap100 C18, 75 µm, 150 mm, 3 µm; Thermo Fisher Scientific Inc.) with a linear gradient of acetonitrile. Ultimate 3000 LC instrument (Thermo Fisher Scientific Inc.) was operated in nano scale with the flow rate of 0.3 µL/min. Eluted peptides were introduced to an LTQ Orbitrap XL mass spectrometer (Thermo Fisher Scientific Inc.) via ESI Chip interface (Advion BioSciences Inc., Ithaca, NY) in a positive ion mode. Data files from MS were processed with Mascot Distiller (Matrix Science Ltd., London, UK, version 2.3.2). The processed data were searched with Mascot Server (Matrix Science Ltd., version 2.3) against human proteins in UniProtKB database (release 2012_08). Subcellular locations for identified proteins were collected from UniProt database (as is 24.9.2012). Gene ontology (GO) enrichment analysis of identified proteins was performed using the Database for Annotation, Visualization and Integrated Discovery (DAVID, version 6.7) (41) using default settings

### Co-culture assays

Peripheral blood mononuclear cells (PBMCs) were isolated from buffy coats from healthy, anonymous blood donors (Finnish Red Cross Blood Service) by density gradient centrifugation (Ficoll-Pague plus, GE Healthcare) and cryo-preserved for later use. hUCBMSCs or MVs were co-cultured with 1.5×10^6^ CFSE [5(6)-carboxyfluorescein diacetate *N*-succinimidyl ester] solution (Molecular Probes, Eugene, OR) labelled PBMCs. After the collection of medium, 1.5×10^5^ trypsinized hUCBMSCs were resuspended in RPMI growth medium (RPMI, 5% FBS, 100 U/mL penicillin and 100 µg/mL streptomycin) and plated (in triplicates) on a 48-well plate. To activate the T-cell proliferation, 100 ng/mL of the antihuman CD3 antibody clone Hit3a (BioLegend, San Diego, CA) was added to the co-culture. MVs were resuspended in RPMI medium and added in 50 µL in volume (in triplicates) into the co-culture just before adding the stimulant. T-cell proliferation was recorded after 4 days of incubation as a dilution of fluorescent dye by flow cytometry (FACSAria, BD) and data were analyzed using the FlowJo (7.6.5, Treestar, Asland, OR).

To analyze the effect of hUCBMSCs and MVs on Treg induction, PBMCs were co-cultured with MVs or hUCBMSCs. After 7 days of incubation, non-adherent PBMCs were harvested and labelled with fluorogenic antibodies, CD4-APC-Cy7 (Biolegend), CD25-AlexaFluor^®^647 (Biolegend), FOXP3-PerCP5.5 (eBioscience, San Diego, CA) to evaluate the proportion of CD4^+^CD25^+^FOXP3^+^ Tregs. Cells were fixed and permeabilized with FOXP3 Staining Buffer Set (eBioscience) according to manufacturer's instructions and including the blocking step with 2% rat serum (eBioscience). PBMCs cultured without MSCs or MVs were used as a control. Appropriate isotype controls were used. Flow cytometry was performed with FACSAria (BD) and data were analyzed using the FlowJo 7.6.5 software (Treestar).

### Kidney ischemia-reperfusion (I/R) injury model

Eighteen 6- to 7-week-old male Sprague Dawley rats were purchased from Charles River Laboratories (Research Models and Services, Sulzfeld, Germany). The protocols were approved by the Animal Experimentation Committee of the University of Helsinki, Finland, and the Provincial State Office of Southern Finland (approval number STH059A), whose standards correspond to those of the American Physiological Society. The rats were kept under 12-hour light/12-hour dark cycle and were divided into 3 groups: (1) I/R group (n=8), (2) I/R group+control MV (n=5), (3) I/R group+IFN-γ-treated MV (n=5). An established model of kidney I/R injury was used (42). The rats were anesthetized with isoflurane (anaesthesia induction in a chamber with 4–5% isoflurane at the flow rate of 1.5 L/min), intubated, and 1.5% isoflurane at the rate of 1.5 L/min was used to maintain anaesthesia. Abdominal incisions were made and the renal pedicles were bluntly dissected. Bilateral renal ischemia was induced by clamping renal pedicles for 40 minutes with microvascular clamps. After reperfusion, MVs dissolved in 0.5 mL PBS were infused into the rats via the left carotid artery. Controls received the same amount of vehicle. The rats were hydrated with warm saline during the operation and the body temperature was maintained constantly at 37°C by using a heating pad until the rats were awake. The wounds were sutured after removing the clips, and the animals were allowed to recover. Buprenorphine (0.1 mg/kg s.c.) was used as post-operative analgesia. Twenty-four hours after the operation, blood samples were collected from the tail vein under short isoflurane anaesthesia. Terminal samples were harvested 48 hours after the operation; the rats were anesthetized with isoflurane, and blood samples were collected from the inferior vena cava with a 5 mL syringe and 22G needle for biochemical measurements. The kidneys were excised, washed with ice-cold saline, blotted dry and weighed. Left kidney was used for histological examinations. Tissue samples for histology were fixed in 10% formaline and processed to paraffin with routine methodology.

### Kidney histology

For histological examination, 4-µm thick paraffin sections were cut and stained with hematoxylin-eosin (n=9–10). Renal samples were visually examined by a pathologist (P. F.) with Leica DMR microscope (Leica Microsystems AG, Heerbrugg, Switzerland) and morphological changes from the whole cross-sectional area of cortex and medulla were assessed according to the acute tubular necrosis (ATN)-scoring system (42) (Magnification×200, ≥20 fields per kidney section quantified using the ATN-scoring system). The evaluation of histopathological changes included the loss of tubular brush border, tubular dilatation, cast formation and cell lysis. Tissue damage was quantified in a blinded manner and scored according to the percentage of damaged tubules in the sample: 0, no damage; 1, less than 12.5% damage; 2, 12.5–25% damage; 3, 25–50% damage; 4, 50–75% damage; and 5, more than 75% damage.

### Biochemical determinations

Serum creatinine, electrolytes, lipids and liver enzymes were measured by routine laboratory techniques (ADVIA 1650 Chemistry System, Siemens Healthcare Diagnostics Inc., Deerfield, IL).

### Statistical analysis

Data are presented as the mean±standard error of the mean (SEM). Statistically significant differences in mean values were tested by one-way analysis of variance (ANOVA) and the Tukey's post-hoc test using statistical programming software R (version 2.13.1). The differences were considered significant when p<0.05.

## Results

### hUCBMSCs produce MVs which increase the percentage of CD25^+^FOXP3^+^ T-cells and suppress T-cell proliferation

**Fig. 1:**
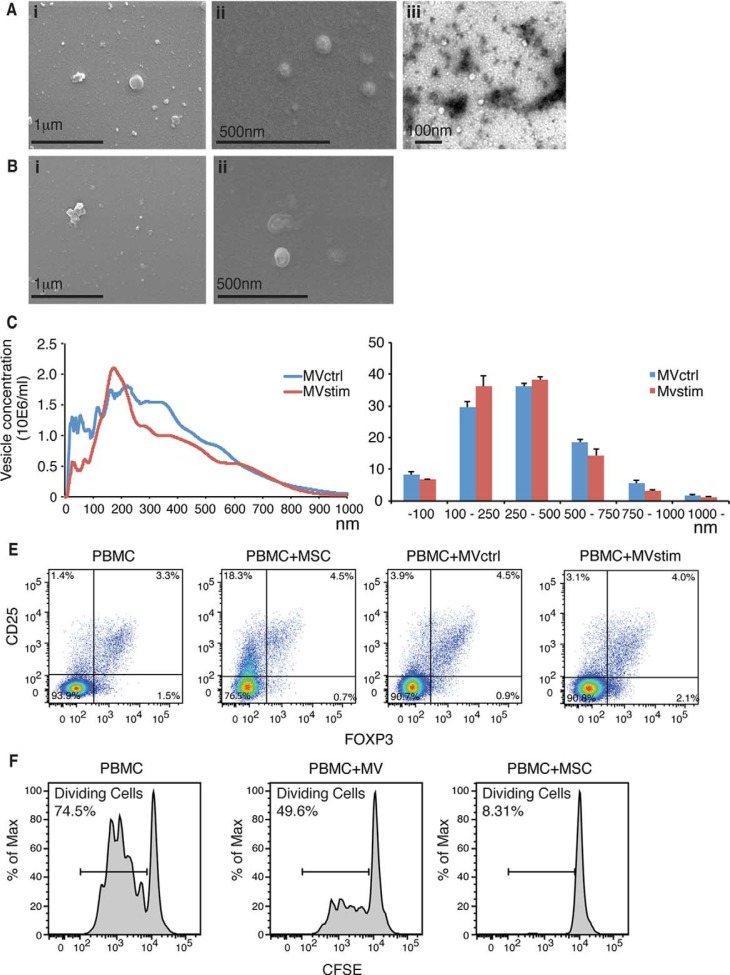
(A) Scanning electron microscopic (FEI Quanta 250 FEG SEM) pictures of MVs produced by serum deprivation at magnification of 60,000× (i) and 90,000× (ii). Negative stainings (FEI Tecnai 12 TEM) of the same sample at magnification of 30,000×(iii). (B) Scanning electron microscopic pictures of MVs produced by IFN-γ stimulation at magnification of 60,000× (i) and 90,000× (ii). (C) Representative nanoparticle tracking analysis (NTA) profiles of MVctrl and MVstim. (D) Size distribution of MV ctrl and MVstim measured by NTA. Results are mean±SEM of 3 independent experiments. (E) The effect of MVs or hUCBMSCs on Treg induction after 7 days of MV or MSC cultured with allogeneic PBMCs. Representative flowcytometric analysis of CD25^+^-FOXP3^+^ Tregs are shown for CD4+ gated T lymphocytes. (F) The effect of MV and MSCs on T-cell proliferation analyzed by CFSE labelling of PBMCs and activation of T-cells with monoclonal CD3 antibody.

hUCBMSCs were induced to produce MVs by culturing them in serum-free conditions with (MVstim) or without (MVctrl) IFN-γ-stimulation for 24–48 hours. Cell viability in both culture conditions was always >95% (n=10). Electron microscopy analysis of the vesicles showed variation in size, with the smallest being around 20 nm and the largest >500 nm (Fig. 1A and B). NTA confirmed the wide size range seen by electron microscopy analysis (Fig. 1C). Also, the presence of very small vesicles (<50 nm) was confirmed. There was no significant difference in the size distribution between IFN-γ stimulated and control conditions (Fig. 1D). Based on the analysis of protein amount, IFN-γ stimulus gave only slightly better MV yield (data not shown). The protein yield was on average 16 µg of protein in a vesicle fraction for every 10×10^6^ cells after 24-h production. When hUCBMSCs or MVs derived from hUCBMSCs were co-cultured with PBMCs for 7 days, we were able to show that MVs alone as well as MSCs were able to induce the formation of T-cells with Treg phenotype (CD4^+^ CD25^+^ FOXP3^+^). The percentage of Tregs was increased from 3.3% to 4.5% when PBMCs were co-cultured with MSCs (Fig. 1E). However, we were not able to see any remarkable differences in Treg induction when PBMCs were co-cultured with either MVctrl (4.5%) or MVstim (4.0%).

Next, we analyzed the ability of hUCBMSCs or the MVs to suppress T-cell proliferation. CFSE labelled PBMCs were cultured with MSCs or MVs. T-cells were activated with antiCD3 monoclonal antibody and the proliferation was analyzed by flow cytometer after 4 days of incubation. We show that both hUCBMSCs and UCBMSC MVs were able to suppress the proliferation of stimulated T-cells, but the MVs alone suppressed T-cell proliferation to a lesser extent (Fig. 1F).

### Protein content of MVs was markedly different after IFN-γ stimulation

**Fig. 2:**
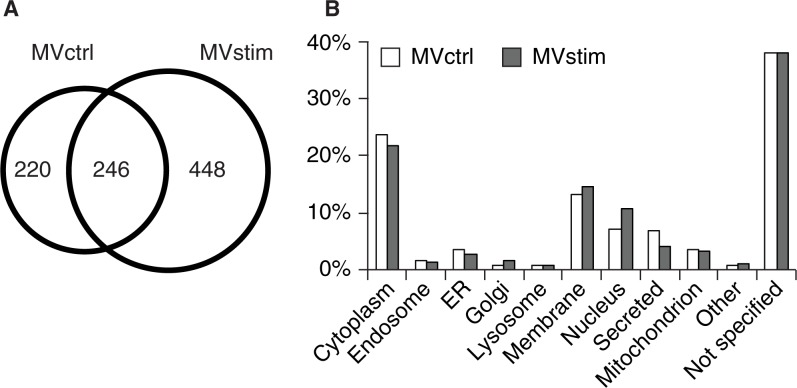
Proteome analysis of hUCBMSC MVs. (A) The Venn diagram illustrates common and unique proteins in control MVs (MVctrl) and stimulated (MVstim). (B) The subcellular locations of protein found in either MVctrl (white bar) or MVstim (grey bar) according to UniprotKB database.

To analyze the protein content of the MVs produced by hUCBMSCs, we performed a gel-based MS analysis of the proteome of secreted MVs with or without IFN-γ stimulation. As demonstrated in a Venn diagram (Fig. 2A), 246 of the proteins were common for both groups, 220 proteins were found only in MVctrl and 448 proteins in MVstim.

MVs contained proteins which originated from different cellular compartments as illustrated in Fig. 2B. Roughly 25% of identified proteins were cytoplasmic, 15% were membrane proteins, and 10% were from the nucleus. Proteins located in endosomes, endoplasmic reticulum and mitochondrion were also identified (Fig. 2B).Altogether, 5–8% of all identified proteins were classified as secreted proteins. The distribution of subcellular localizations of identified proteins from MVctrl and MVstim had only minor differences.

**Fig. 3:**
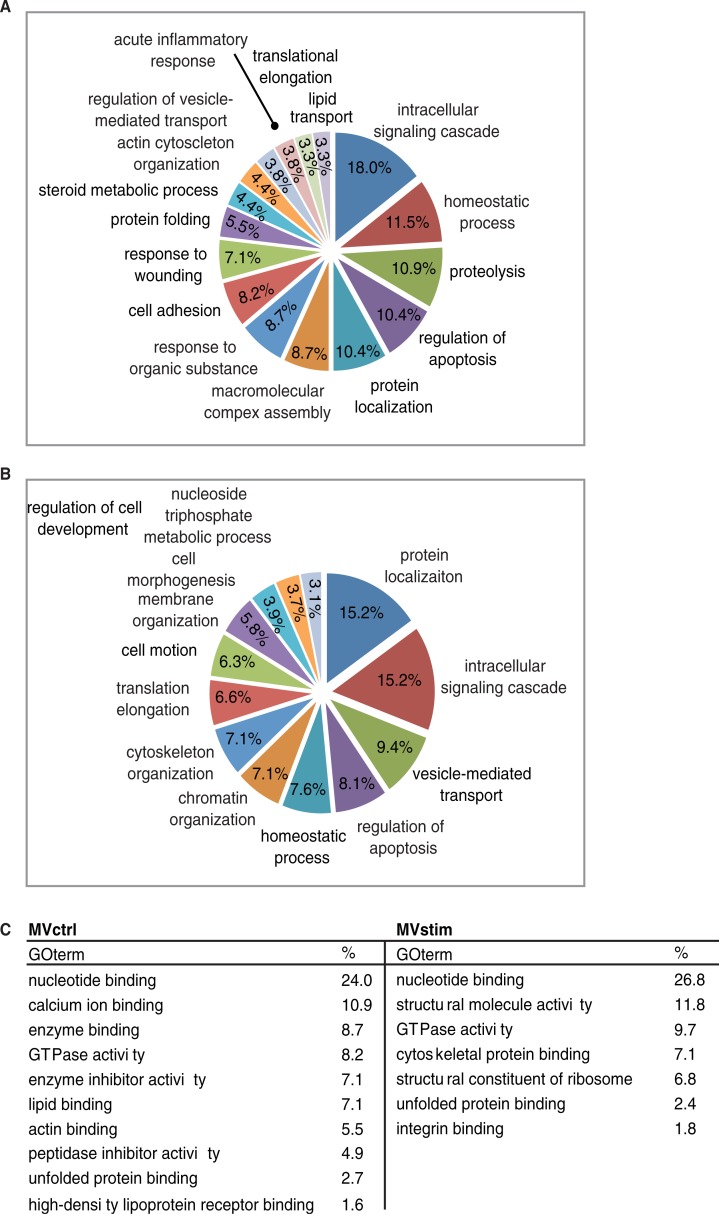
Functional enrichment analysis of MV proteome. (A) Major enriched gene ontology biological processes (GO-PB) terms of 220 proteins identified only in MVctrl. (B) Major enriched GO-PBs of 448 proteins identified only from MVstim. (C) Major GO molecular functions enriched in MVctrl or MVstim. Similar GO terms were excluded for clarity.

The systematic functional GO enrichment analysis was performed by using the DAVID software. Common proteins were excluded from the analysis. Enriched GO biological processes (GO-BP) for MVctrl contained a response to wounding, lipid transport and acute inflammatory response (Fig. 3A). Enriched GO-BP terms unique for MVstim included homeostatic processes, chromatin organization and cell motion (Fig. 3B). Even though proteins subjected to the enrichment analysis were different in both groups, the same GO terms, such as intracellular signalling cascade, regulation of apoptosis and homeostatic processes, were enriched in both groups. This phenomenon was also seen when we analyzed GO molecular functions (GO-MF) (Fig. 1C). GO-MF terms, such as nucleotide binding, GTPase activity and unfolded protein binding, were enriched in both MVs studied.

The detailed MS analysis revealed several differences in the protein content of MVctrl and MVstim. The result of MS analysis was compared to the most often reported proteins from exosomes [Exocarta database as is 16. 4. 2013 (43)]. Altogether, 22 proteins of the top 25 proteins were identified from MVstim compared with only 13 proteins from MVctrl.

Heat-shock protein 70 (HSP70) was found in MVctrl, whereas heatshock protein 90 (HSP90), and mitochondrial heat shock protein 60 (HSP60) were found in both MV groups. Several apolipoproteins (APOA1, APOA2, APOA4 and APOC3) and other lipid-binding proteins (RBP4, SCP2, FABP6) and phospholipase D3 were unique to MVctrl. In addition, several complement-related proteins (C3, C4A, C5 and CD93) were found only in MVctrl.

**Fig. 4:**
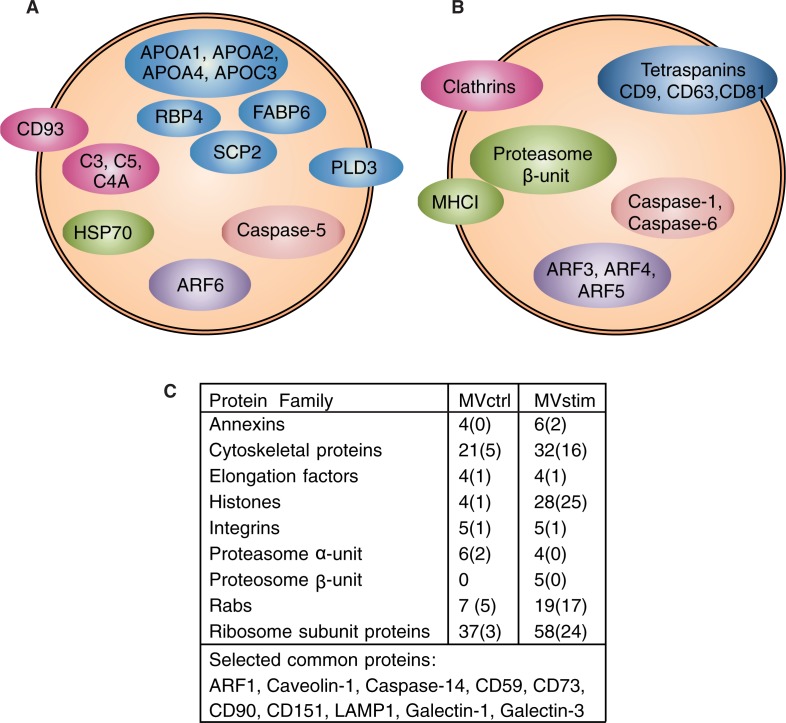
Detailed analysis of MV proteomes revealed remarkable differences in protein compositions of MVctrl and MVstim. Proteins involved in vesicular transport, immunological response and regulation of apoptosis were selected for illustration. (A) Proteins identified by mass spectrometry only from MVctrl. (B) Proteins identified by mass-spectrometry (MS) only from MVstim. (C) The number of identified proteins included in selected protein families found in MVctrl or MVstim. The number of unique proteins is shown in parenthesis. See Supplementary file for complete lists of identified proteins.

According to MS analysis, MVstim (Fig. 4B and C) contained tetraspanins (CD9, CD63 and CD81), which have previously been used as exosome markers (6, 18). Interestingly, MVstim contained also the HLA-A (MHCI) molecule and both α and β units of the proteasome complex (Fig. 4B and C) required for the antigen presentation and activation of T-cells. The MS detection of HLA molecules was supported by flowcytometric analysis on hUCBMSCs. When hUCBMSCs were stimulated with IFN-γ for 24 hours, the expression of HLA-I was clearly induced. In contrast, the HLA-II expression was induced only after 48 hours of stimulation (Supplementary file).

In general, both MVstim and MVctrl (Fig. 4C) contained proteins involved in T-cell regulation (Galectin-1 and -3) and the complement pathway (CD59). MSC markers CD73, CD90 were found in both MVs. Proteins identified only with 1 peptide were excluded from the analysis, including MSC marker CD105. On a protein-family level, several apoptosis regulating proteins (caspases, annexins and histones), cytoskeleton proteins, adhesion proteins (integrins), vesicle trafficking proteins (Ras-related proteins, Rabs and ADP ribosylating factors, ARFs) and ribosome subunits were identified in both groups. Interestingly, the individual proteins were largely different in MVctrl in comparison to MVstim (Fig. 4B). In particular, the number of histones and ribosome subunits was increased in stimulated MVs (Fig. 4C).

### Effects of MV on kidney function and morphology in I/R injury

**Fig. 5:**
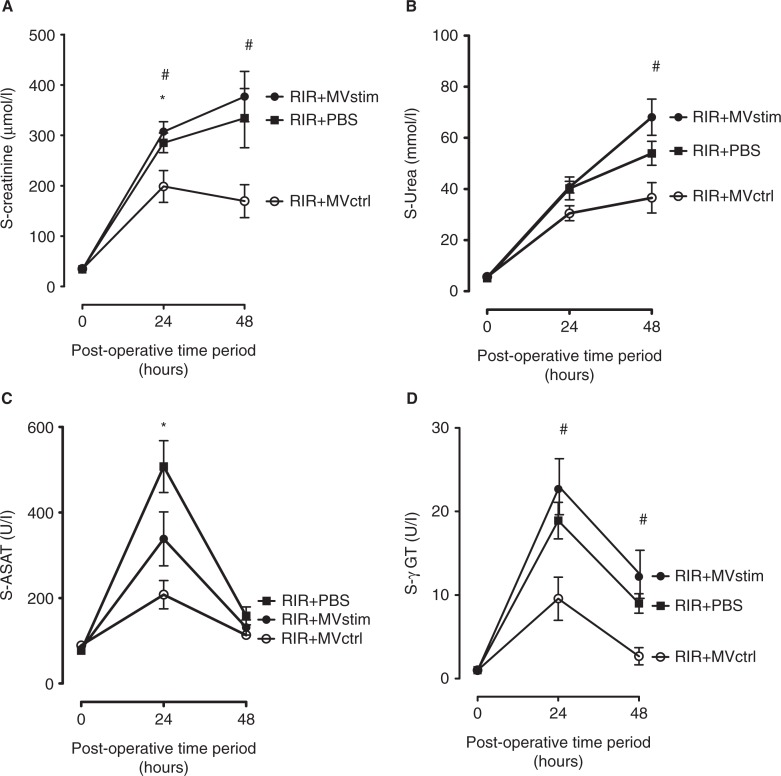
The effects of MV treatment on serum creatinine, urea and liver enzymes in rats with renal ischemia-reperfusion injury measured 24 and 48 hours post-operation. (A) Serum creatinine concentrations (µmol/L). (B) Serum urea concentrations (mmol/L). (C) serum aspartate aminotransferase (ASAT) activity (U/L). (D) Serum gamma glutamyltransferase (γ-GT) activity (U/L). RIR denotes rats with renal I/R injury. RIR+PBS, rats with I/R injury treated with PBS after reperfusion (solid squares); RIR+MVctrl, rats with I/R injury treated with control microvesicles (open circles); RIR+MVstim, rats with I/R injury treated with interferon-γ microvesicles (solid circles). Mean±SEM are given, n=5–8 in each group. *p<0.05 RIR+MVctrl vs. RIR; #p<0.05 RIR+MVctrl vs. RIR+MVstim.

**Fig. 6:**
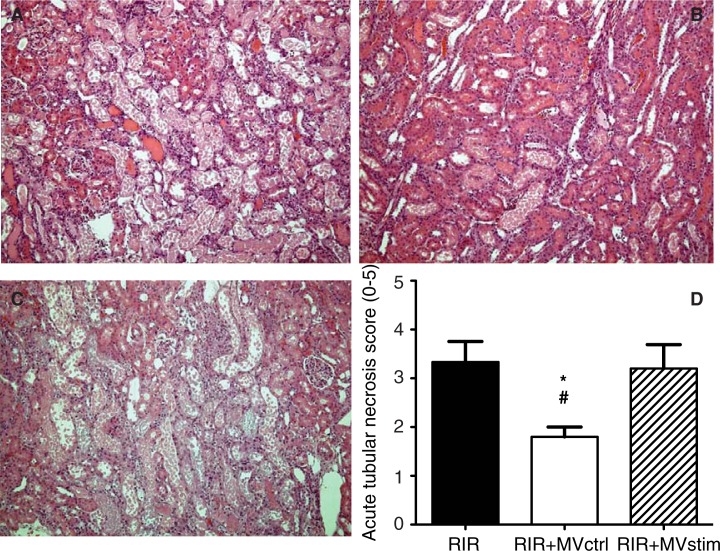
Effects of MV treatment on acute tubular necrosis (ATN) in rats with renal ischemia-reperfusion injury. (A) Representative photomicrographs from untreated rats with I/R injury. (B) Representative photomicrographs from rats with I/R injury treated with MVctrl. (C) Representative photomicrographs from rats with I/R injury treated with MVstim. Magnification ×200, scale bar 100 µm. (D) Quantification of ATN with a scoring system. RIR denotes rats with renal I/R injury; RIR+MVctrl, rats with I/R injury treated with control microvesicles after reperfusion; RIR+MVstim, rats with I/R injury treated with interferon-γ microvesicles after reperfusion; *p<0.05 RIR+MVctrl vs. RIR; #p<0.05 RIR+MVctrl vs. RIR+MVstim.

Acute kidney I/R injury was associated with an 8.4-fold increase in serum creatinine concentration as compared to pre-operative baseline values (Fig. 5A). The concentrations of serum urea also had an 8-fold increase (Fig. 5B) and liver enzyme aspartate aminotransferase (ASAT) had a 6.6-fold increase (Fig. 5C) and gamma glutamyltransferase (γ-GT) had an 18.9-fold increase (Fig. 5D) in 24 hours after operation. Histopathological analysis of the kidneys harvested 48 hours after I/R showed marked injury of the renal parenchyma comprising vast necrosis of the tubuloepithelial cells, tubular dilatation and cast formation (Fig. 6A and D).

MVctrl treatment ameliorated I/R injury-induced kidney dysfunction (Fig. 5) and ATN (Fig. 6B and D), whereas MVstim did not influence the development of kidney I/R injury (Fig. 5 and 6C and D).

## Discussion

The therapeutic potential of MSCs is currently explained by their ability to regulate immune cells through paracrine interactions. The key players in immune response and immunological diseases are T-cells. It has been established that MSCs suppress T lymphocyte activation and proliferation and alter the T-cell population towards an anti-inflammatory profile by increasing the number of regulatory T-cells (44–47) and repressing the differentiation of Th17 cells (48). Inflammatory cytokines, such as IFN-γ have been shown to induce the production of IDO and PGE_2_, the two best described mediators of MSC immunomodulation (49, 50). Recent studies have demonstrated that extracellular vesicles by themselves are capable of modulating T-cell functions and repairing injured tissues (17, 51–54). Although the effects of cytokine stimulus have been in the focus of several studies performed to elucidate the molecular mechanisms of MSCs, the effects of IFN-γ on the MV production have been completely overlooked.

In the present study, we show that hUCBMSCs secrete constitutively extracellular membrane vesicles (MVctrl) capable of significantly attenuating ischemia/reperfusion kidney injury in rats. When MVctrl were administered immediately after reperfusion, the drastic increases in serum creatinine and urea levels caused by hypoxia and concomitant reperfusion were significantly decreased. The protective effect of MVctrl was also seen in the histopathological analysis of the kidneys after 48 hours, where vast necrosis of the tubuloepithelial cells, tubular dilatation and cast formation was mostly absent in MVctrl-treated rats. Our findings are thus in very good agreement with previous studies (16, 17, 25, 55, 56) demonstrating the renoprotective effects of MV during the early phase of AKI. Our results are also in line with a recent paper by Zhou et al., where MVs derived from umbilical cord stem cells protected kidneys from cisplatin-induced injury (18). To investigate the consequence of the inflammatory signals in MV production, we stimulated hUCBMSCs with IFN-γ and compared the therapeutic effect of the MVs produced by the stimulus to the constitutively produced MVs. In comparison to MVctrls, the MVs produced after IFN-γ stimulation (MVstim) showed no protective potential, which was indicated by the similar elevation of serum creatinine and urea levels to those seen in PBS control rats and further demonstrated by the histopathological analysis. Such a difference was not seen *in vitro* potency assays since the co-culture of PBMCs with both MVs gave equal results; both MVstims and MVctrls were able to deliver immunosuppressive effects by enhancing the proliferation of regulatory CD4^+^ CD25^+^ FOXP3^+^ T-cell population (Fig. 1E), a phenomenon also shown by recent studies on bone marrow and cord blood-derived mice stromal cells (53).

In the AKI, activated immune cells accumulating in the kidney can either repair or further aggravate the injury (57). The number of activated T-cells and anti-inflammatory Tregs correlates with the severity of the tissue damage after hypoxia/reperfusion. A decreased number of Tregs leads to an increase in cell death, infiltration of inflammatory cells and a reduction in kidney function (20). Preclinical studies demonstrate that MSCs can either prevent or reverse the kidney injury (58–64). Both MSCs and MVs derived from them have shown to protect kidneys by promoting epithelial cell survival in the damaged tissue (19, 55, 56). Gandolfo and coworkers (23) demonstrated recently using a murine model of kidney I/R injury that Tregs infiltrate into the kidneys starting at 3 days after the initial ischemic injury. This finding suggests that the anti-inflammatory Tregs promote repair during the healing process in the late phase of AKI rather than acting in the acute phase. Innate immune cells including neutrophils, dendritic cells, monocytes/macrophages and natural killer (NK) cells are responsible for the early response to injury. MSCs are known to modulate the innate immunity by affecting the differentiation of dendritic cells and polarization of M1 macrophages to anti-inflammatory M2 macrophages. MSCs also inhibit the proliferation of NK cells (65). Our findings thus support the notion that the protective effects of unstimulated MVs against kidney I/R injury in the early phase are likely to mediated through innate immune system instead of Tregs-dependent mechanisms.

The clear discrepancy between *in vivo* and *in vitro* results encouraged us to analyze the protein content of MVs for possible explanations. The MS analysis verified the presence of several previously described proteins for extracellular vesicles. Common proteins for both MVctrls and MVstims included several cytoskeletal proteins, ribosomal subunits and integrins. As previously described by others, the therapeutic effect of MVs can be mediated by the functional mRNAs and miRNAs (17, 55, 56). We found 34 common ribosomal proteins in both MV pools; in addition to the common proteins, both pools contained several unique ribosomal proteins. The additional common proteins were functionally interesting, such as Galectin-1 and -3, CD90 and CD73. Moreover, both galectin-1 and -3 have proved to be mediators of MSC T-cell immunosuppression (66–68). Galectin-1 is an important regulator of Tregs (69), whereas galectin-3 has been reported to regulate T-cell function by participating in the cell surface receptor activity (66, 67). Two mesenchymal stromal/stem cell markers, CD73 and CD90 have also been associated with the immunosuppressive capacity (70, 71). The expression of CD73 and its ectonuclease activity producing adenosine have been proposed to be important in the functionality of Tregs (72) and recently the role of adenosine signalling was demonstrated to have a beneficial effect in AKI, as it was shown that the pre-treatment of dendritic cells with adenosine receptor 2 (A2R) agonist protected kidneys from ischemia/reperfusion induced injury in mice (73). The role of CD73 in immune regulation has been further demonstrated in mice deficient in CD73 with more severe graft-versus-host disease than the wild-type controls (74).

**Fig. 7:**
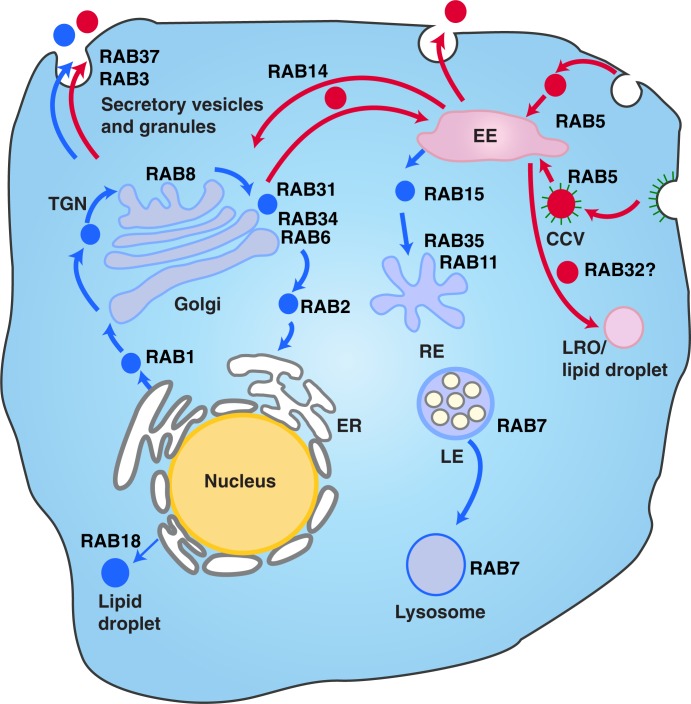
The different vesicle transport pathways and the functions of Rab GTPases identified from the hUCBMSC MVs. Rab proteins found in MVstim were related to deeper endosomal route (blue), whereas MVcrtl contained Rab proteins from the rapid loop located near the plasma membrane (red). RAB1, which is localized at the endoplasmic reticulum (ER), mediates ER–Golgi trafficking together with RAB2, which might also regulate Golgi–ER trafficking. The Golgi-localized RAB6 and RAB34 mediate intra-Golgi trafficking. RAB8 mediates constitutive biosynthetic trafficking from the *trans*-Golgi network (TGN) to the plasma membrane. RAB3, and RAB37 regulate the secretory pathway. RAB32 is involved in the biogenesis of melanosomes and other lysosome-related organelles (LRO) as well as the formation of lipid droplets from the early endosome (EE). RAB18 controls the formation of lipid droplets from the ER. RAB5 mediates endocytosis and endosome fusion of clathrin-coated vesicles (CCVs). RAB11 and RAB35 mediate slow endocytic recycling through recycling endosomes, (RE). RAB15 is involved in the trafficking from early endosomes (EE) to recycling endosomes. The late endosome-associated RAB7 mediates the maturation of late endosomes (LE) and their fusion with lysosomes. See Refs. (75, 79–81) for further information.

There is on-going scientific debate over the cellular origin, characteristics and nomenclature of extracellular vesicles. In this study, we made a conscious decision not to separate the different sizes or densities MVs by ultracentrifugation or filtration processes. Instead, we wanted to collect the whole pool of secreted MVs from the supernatant or hUCBMSCs culture *milieu* and study the effect of the different external signals on the composition of the MVs. One of the most interesting findings in our study was the distinct set of Rab proteins found in the different MV pools produced. It is well established that Rab GTPases, members of the Ras superfamily, are important regulators of membrane transport, vesicle formation, movement and fusion and can be considered as labels defining vesicle routing (75), with also their role in extracellular vesicle formation and transport being studied (76, 77). This superfamily is known to consist of at least 60 members in humans (78). Altogether 24 Rab proteins were identified in our study. The MS analysis revealed that IFN-γ induced production of MVs richer in Rab proteins. Altogether 19 Rab proteins were detected in MVstim, 17 of which were unique. Instead, in MVctrls, only 7 Rab proteins were identified, 5 of which were unique. The Rab proteins found in MVstims were distinctive for exocytosis and deeper endosomal recycling, extending from ER all through the exocytotic pathway (Rab 1, 2, 6 and 8) as illustrated in Fig. 7. In contrast, MVctrls contained Rab proteins more restricted to the early endosomal recycling near plasma membrane (such as Rab 3, 5, 14 and 34). Our interpretation is that this strikingly different and specific subcellular localization of the Rab proteins indicates that for the first time, we have been able to make a distinction between two separate routes of extracellular vesicles from MSCs: cytokine-induced deeper loop and constantly produced rapid loop of MVs. The presence of MHCI molecules in MVstims accompanied by the more complete proteasome complex than in MVctrls further proves the effective IFN-γ activation and the induction of the delivery of MHCI molecules to of the cell surface and consequently into MVstims. Already in 2003, Willem Stoorvogel's group described proteomic analysis of B-cell-derived exosomes (82). They demonstrated that B-cell-derived exosomes contained major histocompatibility Class I and II (MHCI and II) as major components in addition to integrins and many other proteins also seen in MSC-derived MVs. On the contrary to B-cells, MSCs do not express MHCII molecules on their cell surface. Furthermore, MHCI molecules are expressed at low levels. Our results show that after 24 hours of IFN-γ activation of hUCBMSCs, the surface expression of MHCI is increased but not MHCII for which 48 hours of activation is required. Interestingly, we found that MSC-derived MVctrls do not contain either of these molecules but after IFN-γ activation, MHCI and also a complete set of proteasome complex were detected in MVstims.

The presence of the tetraspanin complex (CD9, CD63 and CD81), previously described as a marker of exosomes, in MVstims possibly indicates that this pool of MVs is enriched with extracellular vesicles previously described as exosomes. The functional consequence of the self-antigen presentation (MHCI) in MVstims may entirely or partly explain the loss of function in MVstims in AKI model. It is possible that the presence of the MHCI on the surface of MVstims triggers the innate immune response in rats and although otherwise functional (as seen in human-cell-based co-culture assays), the MVstims might be cleared faster from the circulation and never reach their therapeutic target in adequate numbers. Another explanation might be found in the different content of MVs. We did discover unique and plausible candidates in MVctrls that could explain their enhanced therapeutic potential *in vivo*. Only the MVctrls contained complement activation-related proteins CD59, C5, C3 and C4A, which on their own or via their degradation products are key molecules in inflammatory responses and the initiation of the classical complement pathway (83), which as such is speculated to be important in the clearance of apoptotic and necrotic cells by phagocytic macrophages (84, 85) in damaged tissue.

Further MVctrls contained anti-inflammatory and anti-oxidative apolipoprotein A-I (ApoA1). ApoA1 or high-density lipoproteins (HDL), which major apoliprotein is apoA1, has been shown to protect vascular endothelium in many pathological conditions including atherosclerosis and stroke (86). Recently, the ApoA1 mimetic compound has been reported to have therapeutic effect in kidney injury (87). Furthermore, other apolipoproteins (ApoA2, ApoA4 and ApoC3) and lipid-binding proteins (SCP2 and FABP6) were present only in MVctrls, indicating their likely protective role in AKI. Since it is well known that different apoliproteins and their corresponding lipoprotein particles, containing proteins and lipids, are important in regulation of inflammation and tissue repair, the presence of apolipoproteins only in *in vivo* functional MVctrls may be of special importance.

## Conclusions

Our study compares for the first time the T-cell regulation, therapeutic effect and protein content of differently produced MVs from the same MSC population. The results demonstrate that different external conditions lead to the production of MVs, which originate from very distinct internal vesicle routes, have completely different therapeutic potential and contain unique proteins expected to play a critical role in their function. As MSCs have diverse immunological functions, our results indicate that membrane trafficking routes previously considered as intracellular might actually be an efficient way for the cell to separate diverse extracellular signals and secrete the specific responses as MVs. Further studies are needed to fully elucidate the functional role of the numerous MV proteins identified in this study.

## References

[1] Wolf P (1967). The nature and significance of platelet products in human plasma. Br J Haematol.

[2] Ratajczak J, Wysoczynski M, Hayek F, Janowska-Wieczorek A, Ratajczak MZ (2006). Membrane-derived microvesicles: important and underappreciated mediators of cell-to-cell communication. Leukemia.

[3] György B, Módos K, Pállinger É, Pálóczi K, Pásztói M, Misják P (2011). Detection and isolation of cell-derived microparticles are compromised by protein complexes resulting from shared biophysical parameters. Blood.

[4] Van der Pol E, Boing AN, Harrison P, Sturk A, Nieuwland R (2012). Classification, functions, and clinical relevance of extracellular vesicles. Pharmacol Rev.

[5] Valadi H, Ekstrom K, Bossios A, Sjostrand M, Lee JJ, Lotvall JO (2007). Exosome-mediated transfer of mRNAs and microRNAs is a novel mechanism of genetic exchange between cells. Nat Cell Biol.

[6] Tomasoni S, Longaretti L, Rota C, Morigi M, Conti S, Gotti E (2013). Transfer of growth factor receptor mRNA via exosomes unravels the regenerative effect of mesenchymal stem cells. Stem Cells Dev.

[7] Thery C, Ostrowski M, Segura E (2009). Membrane vesicles as conveyors of immune responses. Nat Rev Immunol.

[8] Le Blanc K, Frassoni F, Ball L, Locatelli F, Roelofs H, Lewis I (2008). Mesenchymal stem cells for treatment of steroid-resistant, severe, acute graft-versus-host disease: a phase II study. Lancet.

[9] Dalal J, Gandy K, Domen J (2012). Role of mesenchymal stem cell therapy in Crohn's disease. Pediatr Res.

[10] MacDonald GIA, Augello A, De Bari C (2011). Role of mesenchymal stem cells in reestablishing immunologic tolerance in autoimmune rheumatic diseases. Arthritis Rheum.

[11] Di Nicola M, Carlo-Stella C, Magni M, Milanesi M, Longoni PD, Matteucci P (2002). Human bone marrow stromal cells suppress T-lymphocyte proliferation induced by cellular or nonspecific mitogenic stimuli. Blood.

[12] English K, Ryan JM, Tobin L, Murphy MJ, Barry FP, Mahon BP (2009). Cell contact, prostaglandin E2 and transforming growth factor beta 1 play non-redundant roles in human mesenchymal stem cell induction of CD4 + CD25Highforkhead box P3+ regulatory T cells. Clin Exp Immunol.

[13] Kong QF, Sun B, Wang GY, Zhai DX, Mu LL, Wang DD (2009). BM stromal cells ameliorate experimental autoimmune myasthenia gravis by altering the balance of Th cells through the secretion of IDO. Eur J Immunol.

[14] Aggarwal S, Pittenger MF (2005). Human mesenchymal stem cells modulate allogeneic immune cell responses. Blood.

[15] Meisel R, Zibert A, Laryea M, Göbel U, Däubener W, Dilloo D (2004). Human bone marrow stromal cells inhibit allogeneic T-cell responses by indoleamine 2,3-dioxygenase-mediated tryptophan degradation. Blood.

[16] He J, Wang Y, Sun S, Yu M, Wang C, Pei X (2012). Bone marrow stem cells-derived microvesicles protect against renal injury in the mouse remnant kidney model. Nephrology.

[17] Gatti S, Bruno S, Deregibus MC, Sordi A, Cantaluppi V, Tetta C (2011). Microvesicles derived from human adult mesenchymal stem cells protect against ischaemia-reperfusion-induced acute and chronic kidney injury. Nephrol Dial Transplant.

[18] Zhou Y, Xu H, Xu W, Wang B, Wu H, Tao Y (2013). Exosomes released by human umbilical cord mesenchymal stem cells protect against cisplatin-induced renal oxidative stress and apoptosis in vivo and in vitro. Stem Cell Res Ther.

[19] Togel FE, Westenfelder C (2010). Mesenchymal stem cells: a new therapeutic tool for AKI. Nat Rev Nephrol.

[20] Okusa MD (2002). The inflammatory cascade in acute ischemic renal failure. Nephron.

[21] Ascon DB, Lopez-Briones S, Liu M, Ascon M, Savransky V, Colvin RB (2006). Phenotypic and functional characterization of kidney-infiltrating lymphocytes in renal ischemia reperfusion injury. J Immunol.

[22] Kinsey GR, Huang L, Vergis AL, Li L, Okusa MD (2010). Regulatory T cells contribute to the protective effect of ischemic preconditioning in the kidney. Kidney Int.

[23] Gandolfo MT, Jang HR, Bagnasco SM, Ko GJ, Agreda P, Satpute SR (2009). Foxp3+ regulatory T cells participate in repair of ischemic acute kidney injury. Kidney Int.

[24] Asanuma H, Meldrum DR, Meldrum KK (2010). Therapeutic applications of mesenchymal stem cells to repair kidney injury. J Urol.

[25] Cantaluppi V, Gatti S, Medica D, Figliolini F, Bruno S, Deregibus MC (2012). Microvesicles derived from endothelial progenitor cells protect the kidney from ischemia-reperfusion injury by microRNA-dependent reprogramming of resident renal cells. Kidney Int.

[26] DelaRosa O, Lombardo E, Beraza A, Mancheno-Corvo P, Ramirez C, Menta R (2009). Requirement of IFN-gamma-mediated indoleamine 2,3-dioxygenase expression in the modulation of lymphocyte proliferation by human adipose-derived stem cells. Tissue Eng Part A.

[27] Ren G, Zhang L, Zhao X, Xu G, Zhang Y, Roberts AI (2008). Mesenchymal stem cell-mediated immunosuppression occurs via concerted action of chemokines and nitric oxide. Cell Stem Cell.

[28] English K, Barry FP, Field-Corbett CP, Mahon BP (2007). IFN-gamma and TNF-alpha differentially regulate immunomodulation by murine mesenchymal stem cells. Immunol Lett.

[29] Ryan JM, Barry F, Murphy JM, Mahon BP (2007). Interferon-gamma does not break, but promotes the immunosuppressive capacity of adult human mesenchymal stem cells. Clin Exp Immunol.

[30] Polchert D, Sobinsky J, Douglas G, Kidd M, Moadsiri A, Reina E (2008). IFN-gamma activation of mesenchymal stem cells for treatment and prevention of graft *versus* host disease. Eur J Immunol.

[31] Krampera M (2011). Mesenchymal stromal cell ‘licensing’: a multistep process. Leukemia.

[32] Sheng H, Wang Y, Jin Y, Zhang Q, Zhang Y, Wang L (2008). A critical role of IFN-gamma in priming MSC-mediated suppression of T cell proliferation through up-regulation of B7-H1. Cell Res.

[33] Bieback K, Kern S, Kocaomer A, Ferlik K, Bugert P (2008). Comparing mesenchymal stromal cells from different human tissues: bone marrow, adipose tissue and umbilical cord blood. Biomed Mater Eng.

[34] Najar M, Raicevic G, Boufker HI, Kazan HF, Bruyn CD, Meuleman N (2010). Mesenchymal stromal cells use PGE2 to modulate activation and proliferation of lymphocyte subsets: combined comparison of adipose tissue, Wharton's Jelly and bone marrow sources. Cell Immunol.

[35] Hsiao ST, Asgari A, Lokmic Z, Sinclair R, Dusting GJ, Lim SY (2012). Comparative analysis of paracrine factor expression in human adult mesenchymal stem cells derived from bone marrow, adipose, and dermal tissue. Stem Cells Dev.

[36] Prasanna SJ, Gopalakrishnan D, Shankar SR, Vasandan AB (2010). Pro-inflammatory cytokines, IFNgamma and TNF-alpha, influence immune properties of human bone marrow and Wharton jelly mesenchymal stem cells differentially. PLoS One.

[37] Laitinen A, Nystedt J, Laitinen S (2011). The isolation and culture of human cord blood-derived mesenchymal stem cells under low oxygen conditions. Methods Mol Biol.

[38] Dominici M, Le Blanc K, Mueller I, Slaper-Cortenbach I, Marini F, Krause D (2006). Minimal criteria for defining multipotent mesenchymal stromal cells. The International Society for Cellular Therapy position statement. Cytotherapy.

[39] O'Connell KL, Stults JT (1997). Identification of mouse liver proteins on two-dimensional electrophoresis gels by matrix-assisted laser desorption/ionization mass spectrometry of in situ enzymatic digests. Electrophoresis.

[40] Shevchenko A, Tomas H, Havlis J, Olsen JV, Mann M (2006). In-gel digestion for mass spectrometric characterization of proteins and proteomes. Nat Protoc.

[41] Huang da W, Sherman BT, Lempicki RA (2009). Systematic and integrative analysis of large gene lists using DAVID bioinformatics resources. Nat Protoc.

[42] Lempiäinen J, Finckenberg P, Levijoki J, Mervaala E (2012). AMPK activator AICAR ameliorates ischaemia reperfusion injury in the rat kidney. Br J Pharmacol.

[43] Mathivanan S, Fahner CJ, Reid GE, Simpson RJ (2012). ExoCarta 2012: database of exosomal proteins, RNA and lipids. Nucleic Acids Res.

[44] Selmani Z, Naji A, Zidi I, Favier B, Gaiffe E, Obert L (2008). Human leukocyte antigen-G5 secretion by human mesenchymal stem cells is required to suppress T lymphocyte and natural killer function and to induce CD4+CD25highFOXP3+ regulatory T cells. Stem Cells.

[45] Ianni MD, Beatrice DP, Ioanni MD, Moretti L, Bonifacio E, Cecchini D (2008). Mesenchymal cells recruit and regulate T regulatory cells. Exp Hematol.

[46] Casiraghi F, Azzollini N, Cassis P, Imberti B, Morigi M, Cugini D (2008). Pretransplant infusion of mesenchymal stem cells prolongs the survival of a semiallogeneic heart transplant through the generation of regulatory T-cells. J Immunol.

[47] Engela AU, Hoogduijn MJ, Boer K, Litjens NHR, Betjes MGH, Weimar W (2013). Human adipose-tissue derived mesenchymal stem cells induce functional de novo regulatory T-cells with methylated FOXP3 gene DNA. Clin Exp Immunol.

[48] Duffy MM, Pindjakova J, Hanley SA, McCarthy C, Weidhofer GA, Sweeney EM (2011). Mesenchymal stem cell inhibition of T-helper 17 cell-differentiation is triggered by cell–cell contact and mediated by prostaglandin E2 via the EP4 receptor. Eur J Immunol.

[49] Ghannam S, Pène J, Torcy-Moquet G, Jorgensen C, Yssel H (2010). Mesenchymal stem cells inhibit human Th17 cell differentiation and function and induce a T regulatory cell phenotype. J Immunol.

[50] Opitz CA, Litzenburger UM, Lutz C, Lanz TV, Tritschler I, Köppel A (2009). Toll-like receptor engagement enhances the immunosuppressive properties of human bone marrow-derived mesenchymal stem cells by inducing indoleamine-2,3-dioxygenase-1 via interferon-beta and protein Kinase R. Stem Cells.

[51] Lai RC, Arslan F, Lee MM, Sze NS, Choo A, Chen TS (2010). Exosome secreted by MSC reduces myocardial ischemia/reperfusion injury. Stem Cell Res.

[52] Xin H, Li Y, Buller B, Katakowski M, Zhang Y, Wang X (2012). Exosome-mediated transfer of miR-133b from multipotent mesenchymal stromal cells to neural cells contributes to neurite outgrowth. Stem Cells.

[53] Mokarizadeh A, Delirezh N, Morshedi A, Mosayebi G, Farshid A, Mardani K (2012). Microvesicles derived from mesenchymal stem cells: potent organelles for induction of tolerogenic signaling. Immunol Lett.

[54] Wu S, Ju G, Du T, Zhu Y, Liu G (2013). Microvesicles derived from human umbilical cord Wharton's Jelly mesenchymal stem cells attenuate bladder tumor cell growth *in vitro* and *in vivo*. PLoS One.

[55] Bruno S, Grange C, Deregibus MC, Calogero RA, Saviozzi S, Collino F (2009). Mesenchymal stem cell-derived microvesicles protect against acute tubular injury. J Am Soc Nephrol.

[56] Collino F, Deregibus MC, Bruno S, Sterpone L, Aghemo G, Viltono L (2010). Microvesicles derived from adult human bone marrow and tissue specific mesenchymal stem cells shuttle selected pattern of miRNAs. PLoS One.

[57] Bonventre JV, Yang L (2011). Cellular pathophysiology of ischemic acute kidney injury. J Clin Invest.

[58] Alexandre CS, Volpini RA, Shimizu MH, Sanches TR, Semedo P, di Jura VL (2009). Lineage-negative bone marrow cells protect against chronic renal failure. Stem Cells.

[59] Herrera MB, Bussolati B, Bruno S, Morando L, Mauriello-Romanazzi G, Sanavio F (2007). Exogenous mesenchymal stem cells localize to the kidney by means of CD44 following acute tubular injury. Kidney Int.

[60] Behr L, Hekmati M, Lucchini A, Houcinet K, Faussat AM, Borenstein N (2009). Evaluation of the effect of autologous mesenchymal stem cell injection in a large-animal model of bilateral kidney ischaemia reperfusion injury. Cell Prolif.

[61] Perico N, Casiraghi F, Introna M, Gotti E, Todeschini M, Cavinato RA (2011). Autologous mesenchymal stromal cells and kidney transplantation: a pilot study of safety and clinical feasibility. Clin J Am Soc Nephrol.

[62] Ninichuk V, Gross O, Segerer S, Hoffmann R, Radomska E, Buchstaller A (2006). Multipotent mesenchymal stem cells reduce interstitial fibrosis but do not delay progression of chronic kidney disease in collagen4A3-deficient mice. Kidney Int.

[63] Lindoso RS, Araujo DS, Adao-Novaes J, Mariante RM, Verdoorn KS, Fragel-Madeira L (2011). Paracrine interaction between bone marrow-derived stem cells and renal epithelial cells. Cell Physiol Biochem.

[64] Hu J, Zhang L, Wang N, Ding R, Cui S, Zhu F (2013). Mesenchymal stem cells attenuate ischemic acute kidney injury by inducing regulatory T cells through splenocyte interactions. Kidney Int.

[65] Le Blanc K, Mougiakakos D (2012). Multipotent mesenchymal stromal cells and the innate immune system. Nat Rev Immunol.

[66] Lepelletier Y, Lecourt S, Renand A, Arnulf B, Vanneaux V, Fermand JP (2010). Galectin-1 and semaphorin-3A are two soluble factors conferring T-cell immunosuppression to bone marrow mesenchymal stem cell. Stem Cells Dev.

[67] Gieseke F, Bohringer J, Bussolari R, Dominici M, Handgretinger R, Muller I (2010). Human multipotent mesenchymal stromal cells use galectin-1 to inhibit immune effector cells. Blood.

[68] Sioud M, Mobergslien A, Boudabous A, Floisand Y (2011). Mesenchymal stem cell-mediated T cell suppression occurs through secreted galectins. Int J Oncol.

[69] Garín MI, Chu C, Golshayan D, Cernuda-Morollón E, Wait R, Lechler RI (2007). Galectin-1: a key effector of regulation mediated by CD4 + CD25+ T cells. Blood.

[70] Sattler C, Steinsdoerfer M, Offers M, Fischer E, Schierl R, Heseler K (2011). Inhibition of T-cell proliferation by murine multipotent mesenchymal stromal cells is mediated by CD39 expression and adenosine generation. Cell Transplant.

[71] Campioni D, Rizzo R, Stignani M, Melchiorri L, Ferrari L, Moretti S (2009). A decreased positivity for CD90 on human mesenchymal stromal cells (MSCs) is associated with a loss of immunosuppressive activity by MSCs. Cytometry B Clin Cytom.

[72] Deaglio S, Dwyer KM, Gao W, Friedman D, Usheva A, Erat A (2007). Adenosine generation catalyzed by CD39 and CD73 expressed on regulatory T cells mediates immune suppression. J Exp Med.

[73] Li L, Huang L, Vergis AL, Ye H, Bajwa A, Narayan V (2010). IL-17 produced by neutrophils regulates IFN-gamma-mediated neutrophil migration in mouse kidney ischemia-reperfusion injury. J Clin Invest.

[74] Tsukamoto H, Chernogorova P, Ayata K, Gerlach UV, Rughani A, Ritchey JW (2012). Deficiency of CD73/ecto-5′-nucleotidase in mice enhances acute graft-versus-host disease. Blood.

[75] Stenmark H (2009). Rab GTPases as coordinators of vesicle traffic. Nat Rev Mol Cell Biol.

[76] Pfeffer SR (2010). Two Rabs for exosome release. Nat Cell Biol.

[77] Ostrowski M, Carmo NB, Krumeich S, Fanget I, Raposo G, Savina A (2010). Rab27a and Rab27b control different steps of the exosome secretion pathway. Nat Cell Biol.

[78] Hutagalung AH, Novick PJ (2011). Role of Rab GTPases in membrane traffic and cell physiology. Physiol Rev.

[79] Bultema JJ, Di Pietro SM (2013). Cell type-specific Rab32 and Rab38 cooperate with the ubiquitous lysosome biogenesis machinery to synthesize specialized lysosome-related organelles. Small GTPases.

[80] Wang C, Liu Z, Huang X (2012). Rab32 is important for autophagy and lipid storage in *Drosophila*. PLoS One.

[81] Goldenberg NM, Grinstein S, Silverman M (2007). Golgi-bound Rab34 is a novel member of the secretory pathway. Mol Biol Cell.

[82] Wubbolts R, Leckie RS, Veenhuizen PT, Schwarzmann G, Mobius W, Hoernschemeyer J (2003). Proteomic and biochemical analyses of human B cell-derived exosomes. Potential implications for their function and multivesicular body formation. J Biol Chem.

[83] Kim CH, Wu W, Wysoczynski M, Abdel-Latif A, Sunkara M, Morris A (2012). Conditioning for hematopoietic transplantation activates the complement cascade and induces a proteolytic environment in bone marrow: a novel role for bioactive lipids and soluble C5b-C9 as homing factors. Leukemia.

[84] Mevorach D, Mascarenhas JO, Gershov D, Elkon KB (1998). Complement-dependent clearance of apoptotic cells by human macrophages. J Exp Med.

[85] Gullstrand B, Martensson U, Sturfelt G, Bengtsson AA, Truedsson L (2009). Complement classical pathway components are all important in clearance of apoptotic and secondary necrotic cells. Clin Exp Immunol.

[86] Tran-Dinh A, Diallo D, Delbosc S, Varela-Perez LM, Dang Q, Lapergue B (2013). HDL and endothelial protection. Br J Pharmacol.

[87] Shi N, Wu MP (2008). Apolipoprotein A-I attenuates renal ischemia/reperfusion injury in rats. J Biomed Sci.

